# A rare giant pericardial cyst mimicking a paracardiac mass

**DOI:** 10.5830/CVJA-2016-016

**Published:** 2016

**Authors:** Hakan Akbayrak, Serkan Yildirim, Simsek Murat, Oc Mehmet

**Affiliations:** Department of Cardiovascular Surgery, Faculty of Medicine, Selcuk University, Konya, Turkey; Department of Cardiovascular Surgery, Faculty of Medicine, Selcuk University, Konya, Turkey; Department of Cardiovascular Surgery, Faculty of Medicine, Selcuk University, Konya, Turkey; Department of Cardiovascular Surgery, Faculty of Medicine, Selcuk University, Konya, Turkey

**Keywords:** pericardial cyst, surgical treatment, sternotomy

## Abstract

Pericardial cysts are rare benign lesions of the thoracic cavity and are mostly congenital anomalies. They are induced by an incomplete coalescence of foetal lacunae during the development of the pericardium. Pericardial cysts are usually unilocular, well marginated spherical or teardrop shaped and may be attached to the pericardium directly or by a pedicle. Of all pericardial cysts, 70 to 75% are located at the right cardiophrenic angle. We report a case that was incidentally diagnosed with only chest magnetic resonance imaging because of a paracardiac mass. In order to prevent complications, the giant pericardial cyst was excised outside of the pericardium with median sternotomy

## Abstract

Pericardial cysts occur infrequently, with an incidence of one in 100 000 individuals. Mostly they are congenital anomalies but may also be acquired pericardial anomalies (e.g. postinflammatory, hydatid, neoplastic). Pericardial cysts are induced by an incomplete coalescence of foetal lacunae during the development of the pericardium.^1^,^2^

Pericardial cysts are usually unilocular, well marginated spherical or teardrop shaped and may be attached to the pericardium directly or by a pedicle.^3^ Of all pericardial cysts, 70 to 75% are located at the right cardiophrenic angle, and the rest are on the left side of the mediastinum.^4^ They contain clear serous fluid that is called ‘spring water’.^5^ Histologically, these cysts contain a single layer of mesothelial cells, with the remainder of the wall composed of connective tissue with collagen and elastic fibres.^6^

Occasionally, pericardial cysts may alter cardiovascular haemodynamics and/or pulmonary expansion, producing signs and symptoms mimicking tricuspid stenosis, pulmonary stenosis or constrictive pericarditis.^7^ Pericardial cysts occur most frequently in the third or fourth decade of life and the incidence of cases is equal in men and women.^8^

We report on a patient with an extremely large pericardial cyst that was connected to the right atrium. In order to prevent complications, it was excised outside the pericardium with median sternotomy.

## Case report 

**Fig. 1:**
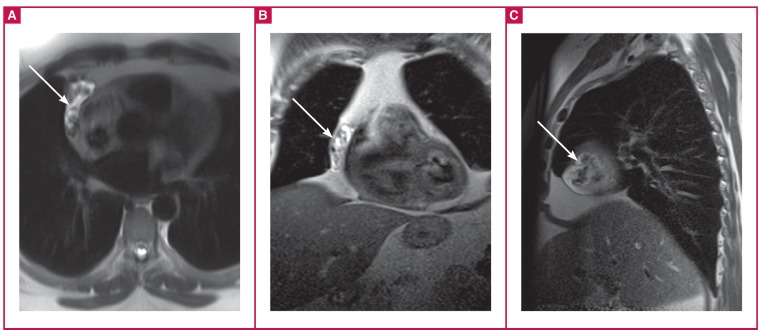
Axial (A), coronal (B) and sagittal (C) views of T2-weighted images in the paracardiac area showing a heterogeneous hyperintense mass (arrows).

A 48-year-old man presented with a dry cough. He was incidentally diagnosed with a paracardiac mass with chest magnetic resonance imaging and referred to our hospital. In the right thoracic cavity, there was a heterogenous hyper-intense mass, which seemed to be connected with the right atrium (Fig. 1). The mass extended from the superior to the inferior vena cavae, outside the pericardium, in the right thoracic cavity.

On examination, his vital signs were as follows: pulse 96 beats/min, blood pressure 140/80 mmHg, body temperature 37.2°C, respiratory rate 18 /min and oxygen saturation on room air was 97%. The white blood cell count was 7 000 cells/μl. His respiratory and heart sounds were normal. The electrocardiogram (ECG) and dobutamine stress echocardiography were normal. Two-dimensional echocardiography from the subcostal view showed a giant homogenous hypo-echoic mass that extended from the superior to the inferior vena cavae.

**Fig. 2:**
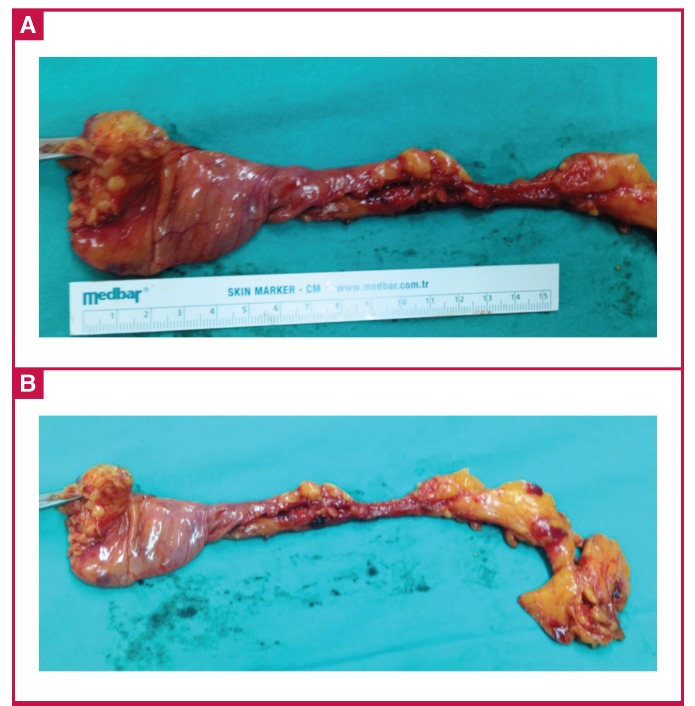
Image of the excised cystic mass

Because of the concern that the mass was connected with the right atrium, we operated on the patient with a median sternotomy and excised the giant paracardiac cystic mass outside the pericardium. The mass measured 27 × 5 × 2 cm (Fig. 2), was well marginated and teardrop shaped, and was attached to the right superior-lateral pericardium by a pedicle.

Pathological examination confirmed the diagnosis of a fibrolipomatous cyst wall with no evidence of malignancy or tissue other than pericardium. There were no other complications in the postoperative period. Our patient was discharged on the seventh day postoperatively.

## Discussion

Pericardial cysts are rare, mostly benign, congenital lesions of the mediastinum, but they may also be acquired pericardial anomalies (e.g. post-inflammatory, hydatid, neoplastic).^1^,^2^ They occur most frequently in the third or fourth decade of life, and occur equally among men and women. The cysts range in size from 2–3 cm, up to a maximum of 28 cm reported by Braude et al.^8^ In our case, the pericardial cyst measured 27 × 5 × 2 cm.

Most of these cases, including ours, are asymptomatic and are diagnosed incidentally on chest X-ray.^4^ The absence of symptoms at diagnosis is a good prognostic sign. However, patients may be admitted to a hospital with symptoms of chest discomfort or pain, cough, dyspnoea, or palpitation due to compression of the heart.^9^,^10^

Life-threatening complications, including cardiac tamponade, obstruction of the right main stem bronchus, cyst infection with cardiac or large vessel erosion and sudden death may be encountered. Cardiac tamponade generally occurs due to intrapericardial rupture of the cyst. Spontaneous cyst rupture and significant haemorrhage into the cysts have been reported, but these have not been linked adversely to cyst size. Asymptomatic cysts of this size are an unusual phenomenon.^11^ Other reported complications include right ventricular outflow tract obstruction, pulmonary stenosis, atrial fibrillation, congestive heart failure, and even sudden death after a stress test.^12^-^14^

Pericardial cysts usually follow a benign course in the majority of cases. There are no reports of malignant transformation.

For asymptomatic patients, conservative management with short follow-up periods is recommended.^15^ Treatment is needed when symptoms or complications occur, and the management of those patients should be performed in the light of clinical characteristics. Indications for surgical resection of pericardial cysts include large size, symptoms, cyst infection, patient request, suspected malignancy, and prevention of complications.^10^,^14^,^16^ Other treatment options for pericardial cysts include simple observation, excision by thoracotomy, thoracoscopic surgical removal, and percutaneous aspiration with injection of a sclerosing agent. Although our patient was asymptomatic, surgical excision was planned due to the large size of the cyst and the concern that the mass was connected to the right atrium.

## Conclusion

Conservative management with short follow-up periods is recommended for asymptomatic patients with pericardial cysts. However, surgery should be considered for patients who become symptomatic and there is doubt about the paracardiac mass. Our patient was unusual because of a rare giant pericardial cyst mimicking a paracardiac mass.
